# Correlation of increased CXCL13 with intrathecal humoral immune responses to HTLV-1 in CSF of patients with HAM/TSP

**DOI:** 10.1186/1742-4690-11-S1-O38

**Published:** 2014-01-07

**Authors:** Yoshimi Enose-Akahata, Raya Massoud, Jussi O Virtanen, Steven Jacobson

**Affiliations:** 1 Viral Immunology Section, Neuroimmunology Branch, National Institute of Neurological Disorders and Stroke, National Institutes of Health, Bethesda, MD, USA

## 

Intrathecal antibody synthesis is a well-documented phenomenon in infectious neurological diseases as well as in demyelinating diseases. Intrathecal antibody synthesis against HTLV-1 has been reported in HAM/TSP, but little is known about the role of B cells and humoral immune responses in the central nervous systems (CNS) of HAM/TSP patients. Here we demonstrate profiles of HTLV-1-specific antibodies in cerebrospinal fluid (CSF) of HAM/TSP patients. Of 36 HAM/TSP patients, antibody responses against Gag and Tax were detected in CSF of all the patients. CSF/Serum antibody ratio was elevated in anti-Gag (mean 1.20) more than in anti-Tax (mean 0.85), but importantly HAM/TSP patients with lesions or atrophy in spinal cord showed higher CSF/Serum anti-Gag antibody ratio. Antibody response against Env was detected in CSF of 94.4% of patients, but CSF/serum anti-Env ratio was significantly lower than those of anti-Gag and anti-Tax (mean 0.18). 19 patients were further studied for oligoclonal band (OCB) specificity to HTLV-1 antigens and all of them were found to have bands specific for at least one antigen studied. Significantly higher proportion of patients had Gag- or Env-specific OCBs than Tax-specific OCBs. Interestingly, CXCL13 (B cell attracting chemokine-1) was increased in CSF of HAM/TSP patients, which was associated with higher HTLV-1-specific antibody responses in CSF and was correlated with decrease of plasma blasts in peripheral blood. These results highlight the importance of the B cell compartment in HAM/TSP where production of HTLV-1-specific antibody may be required to control viral persistence and/or may be associated with HAM/TSP disease progression.

